# Research on a Unified Multi-Type Defect Detection Method for Lithium Batteries Throughout Their Entire Lifecycle Based on Multimodal Fusion and Attention-Enhanced YOLOv8

**DOI:** 10.3390/s26020635

**Published:** 2026-01-17

**Authors:** Zitao Du, Ziyang Ma, Yazhe Yang, Dongyan Zhang, Haodong Song, Xuanqi Zhang, Yijia Zhang

**Affiliations:** 1 School of Civil and Transportation Engineering, Hebei University of Technology, Tianjin 300401, China; 2 School of International Education, Hebei University of Technology, Tianjin 300401, China; 234712@stu.hebut.edu.cn (Z.M.); 244923@stu.hebut.edu.cn (X.Z.); 3 School of Mechanical Engineering, Hebei University of Technology, Tianjin 300401, China; 246058@stu.hebut.edu.cn; 4 School of Electronics and Information Engineering, Hebei University of Technology, Tianjin 300401, China; 230851@stu.hebut.edu.cn; 5 School of Energy and Environmental Engineering, Hebei University of Technology, Tianjin 300401, China; 230986@stu.hebut.edu.cn; 6 School of Materials Science and Engineering, Hebei University of Technology, Tianjin 300401, China; 235666@stu.hebut.edu.cn

**Keywords:** lithium battery defect detection, multimodal fusion, attention mechanism, SE module, multiscale fusion

## Abstract

To address the limitations of traditional lithium battery defect detection—low efficiency, high missed detection rates for minute/composite defects, and inadequate multimodal fusion—this study develops an improved YOLOv8 model based on multimodal fusion and attention enhancement for unified full-lifecycle multi-type defect detection. Integrating visible-light and X-ray modalities, the model incorporates a Squeeze-and-Excitation (SE) module to dynamically weight channel features, suppressing redundancy and highlighting cross-modal complementarity. A Multi-Scale Fusion Module (MFM) is constructed to amplify subtle defect expression by fusing multi-scale features, building on established feature fusion principles. Experimental results show that the model achieves an mAP@0.5 of 87.5%, a minute defect recall rate (MRR) of 84.1%, and overall industrial recognition accuracy of 97.49%. It operates at 35.9 FPS (server) and 25.7 FPS (edge) with end-to-end latency of 30.9–38.9 ms, meeting high-speed production line requirements. Exhibiting strong robustness, the lightweight model outperforms YOLOv5/7/8/9-S in core metrics. Large-scale verification confirms stable performance across the battery lifecycle, providing a reliable solution for industrial defect detection and reducing production costs.

## 1. Introduction

With the rapid development of new energy vehicles and energy storage systems, lithium batteries, as core energy storage components, are drawing increasing attention to their safety and reliability [1]. Throughout their entire lifecycle, specifically encompassing four core stages—electrode manufacturing, cell assembly, aging test, and field service—lithium batteries develop stage-specific defects due to distinct manufacturing processes, environmental stresses, and operational loads. Each stage exhibits unique inspection contexts and defect priors. These stage-dependent defects not only compromise battery performance but also pose potential safety hazards [2], highlighting the need for a unified detection model that adapts to diverse inspection contexts.Consequently, lithium batteries require high-precision, real-time multi-type defect detection [3]. However, traditional inspection methods often rely on manual labor or single-modality imaging [4], which face critical operational bottlenecks in industrial production. For manual inspection, the single-cell inspection cycle averages 12–15 s, severely failing to meet the mass production line requirement of 2–3 cells per second, while the average miss rate for micro-defects is high. These approaches suffer from low efficiency and high rates of missed defects, reaching 18–25%. Existing single-modality AOI (Automated Optical Inspection) systems excel at surface defect detection but have a miss rate of 25–35% for internal defects such as internal short-circuit points and electrode misalignment; single X-ray systems can penetrate to detect internal structures but achieve an accuracy of less than 60% for surface defects like shell scratches and tab deformation [5]. The most frequently overlooked defects are those with pixel ratios of 1–5% and composite defects(cross-stage), which easily evolve into safety hazards such as thermal runaway during battery operation. Additionally, the inspection cycle of single-modality systems (3–5 s per cell) still cannot match the beat of high-speed production lines (≤1 s per cell), leading to production line bottlenecks.

In recent years, research on industrial defect detection and multimodal image analysis has rapidly increased, with extensive work including object detection based on convolutional neural networks (CNNs), semantic segmentation, and cross-modal fusion [6,7]. While semantic segmentation algorithms excel at pixel-level boundary refinement, they face critical limitations in defect detection scenarios, especially for diverse defect granularity and industrial real-time requirements. In contrast, YOLOv8 is uniquely suited for defect granularity and outperforms segmentation algorithms in empirical performance [8]. Overall, existing research can be categorized into several main approaches, summarized as follows. Regarding monomodal object detection and segmentation methods, much work directly applies general-purpose detection or segmentation networks to defect detection [9]. While these methods are simple to implement and train efficiently, their performance is limited when encountering subtle, hidden defects or scenarios where X-ray and visible-light information complement each other, with false negatives and false positives remaining significant issues [10]. Regarding multimodal fusion methods, a rapidly growing research direction involves integrating multiple imaging modalities—such as visible light, X-ray, infrared, or ultrasound—to complement the limitations of single modalities. Several studies have attempted fusion at the data, feature, or decision levels, demonstrating potential for enhancing detection robustness in complex scenarios [11,12]. However, existing fusion strategies often lack unified, universal frameworks and frequently struggle to balance real-time performance with model lightweighting. Attention mechanisms and feature enhancement: To address challenges with minute targets and background interference, numerous studies have introduced multi-scale feature pyramids and feature enhancement structures to boost small-object detection rates [13,14]. Yet their effectiveness hinges on sufficiently diverse training samples and well-designed modules. Regarding end-to-end and lightweight model optimization, to meet industrial online inspection demands for real-time performance and resource-constrained environments, researchers often combine structural modifications with training-level techniques to improve inference speed while maintaining high accuracy [15]. Nevertheless, performance degradation persists in scenarios involving extremely small objects or cross-modal contexts [16]. Synthesizing the above research, multiple productive technical pathways have emerged in this field, achieving significant progress on several representative tasks. Nevertheless, several key challenges persist in the field today: the inability to achieve simultaneous fusion of multimodal information; immature mechanisms for multimodal and multi-scale representation as well as fusion, which makes it challenging to strike a balance between high accuracy and real-time performance, and a lack of unified detection frameworks that cover the entire lifecycle and various defect types. For baseline comparisons, representative detection frameworks are clearly defined: RetinaNet (with ResNet-50 backbone) serves as the non-YOLO industrial baseline, while YOLOv5s, YOLOv7, YOLOv8s, and YOLOv9-S represent mainstream one-stage detectors for performance benchmarking.

This study proposes an improved YOLOv8 approach based on multimodal fusion and attention enhancement, aiming to achieve unified detection of multiple defect types throughout the entire lifecycle of lithium batteries. The core motivation is that strengthening a single-modality YOLO model cannot break through the physical limitations of the modality itself: visible light can capture surface texture details but cannot penetrate the battery shell to identify internal structural defects, while X-ray can reveal internal discontinuities but lacks sensitivity to surface texture features. This makes it impossible to simultaneously address the industrial pain points of high miss rates for cross-type defects and low inspection efficiency. In contrast, combining visible-light (surface defect recognition) and X-ray (internal defect detection) modalities is essential to achieve full-defect coverage. The Squeeze-and-Excitation (SE) module further solves the problem of unbalanced information importance between modalities by dynamically weighting channel features, suppressing redundant information in single modalities, and highlighting complementary features. The MFM targets the key pain point of micro-defect miss detection by fusing multi-scale features to amplify the expression of minute defects (1–5% pixel ratio) that are easily lost in single-modality subsampling. Only the synergistic effect of dual modalities and these two modules can comprehensively solve the bottlenecks of low efficiency, high miss rates, and poor multi-defect adaptability in existing industrial inspection, which is unattainable by simply enhancing a single-modality YOLO model. Specifically, this study constructs a multimodal dataset by integrating visible-light and X-ray image information to overcome the limitations of single-modal images in capturing defect details.It introduces a channel attention mechanism (SE module) to adaptively filter and weight critical information, thereby enhancing detection accuracy. A Multi-Scale Fusion Module (MFM) is designed to amplify the expression of minute defect features. While ensuring real-time performance, the approach further improves detection precision and recall rates. Through the synergistic effects of multimodal fusion and attention enhancement, the improved model effectively boosts detection performance across diverse defect types and scales while maintaining a lightweight architecture and real-time capability. This research will help reduce the risk of missed defects in lithium battery production, enhance safety, minimize redundant costs, and promote the adoption of related technologies in other industrial inspection scenarios.

## 2. YOLOv8 Network and Improvement Methods

### 2.1. YOLOv8 Model Structure

YOLOv8 is a next-generation object detection model released by Ultralytics in 2023. It builds upon the YOLO series’ core strengths of real-time efficiency while achieving breakthroughs in performance and functionality [17]. Its modular network architecture comprises a backbone, neck, and head. The head innovatively employs an anchor-free design, directly predicting the center point, width, height, and category of objects. This reduces reliance on prior boxes while enhancing detection accuracy for small objects [18]. Beyond basic object detection, the model extends to multi-task capabilities including image segmentation and pose estimation. It outperforms predecessor models like YOLOv5 and YOLOv7 on datasets such as COCO [18,19].

Leveraging the ecosystem support of the Ultralytics library, YOLOv8 offers exceptional ease of use. It provides pre-trained models in various scales—n, s, m, l, and x—supports rapid Python 3.13.0 invocation and transfer learning, and can be deployed on edge devices or cloud servers via frameworks like TensorRT and ONNX [17,18]. This makes it widely applicable in scenarios demanding high real-time performance, such as smart surveillance, autonomous driving, and industrial quality inspection. It has emerged as a mainstream computer vision model that effectively balances accuracy, speed, and practicality [20,21].

**Figure 1:**
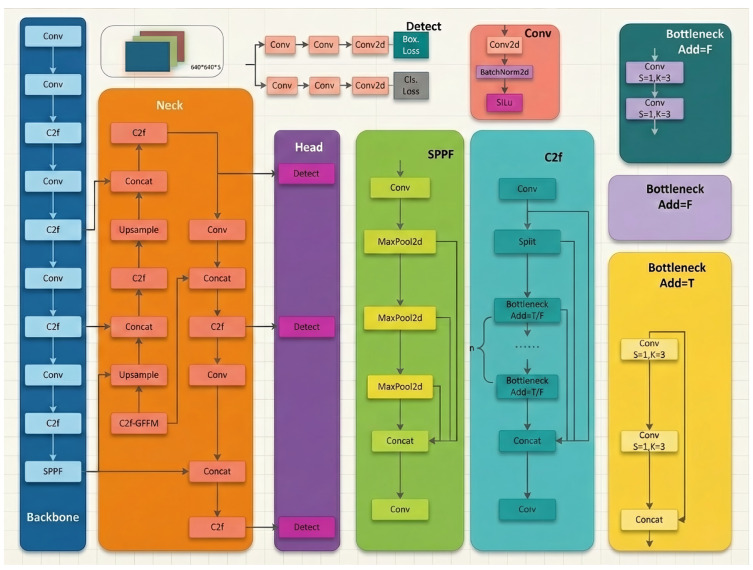
YOLOv8 model diagram.

The architecture of this object detection model is built around the classical paradigm for deep learning object detection tasks. It consists of an input module, basic component layers, a backbone network, a neck network, and a head network working in concert, as shown in Figure 1. Input adapts to dual fusion modes: (1) For feature-level concatenation, the input consists of two separate 640 × 640 resolution images (3-channel RGB visible light + 1-channel X-ray, normalized independently), with features concatenated after the C2f module. (2) For channel-level fusion, the input is a single 640 × 640 4-channel tensor (3 RGB channels + 1 X-ray channel, treated as a unified input). The base component serves as an “atomic unit” supporting feature transformation: the convolution layer (Conv) employs sliding kernels to achieve channel and size transformations while extracting fundamental features—for the 4-channel input scenario, the first convolution layer is adaptively modified to match the input channel dimension, as detailed in Section 3.1.

### 2.2. Squeeze-and-Excitation (SE) Module

The Squeeze-and-Excitation (SE) module, as a pivotal channel attention mechanism in deep learning, plays a crucial role in feature optimization.Its operation follows the ordered logic of Squeeze–Excitation–Scale to achieve adaptive weighting control over the channel dimension of feature maps [22,23].

**Figure 2:**
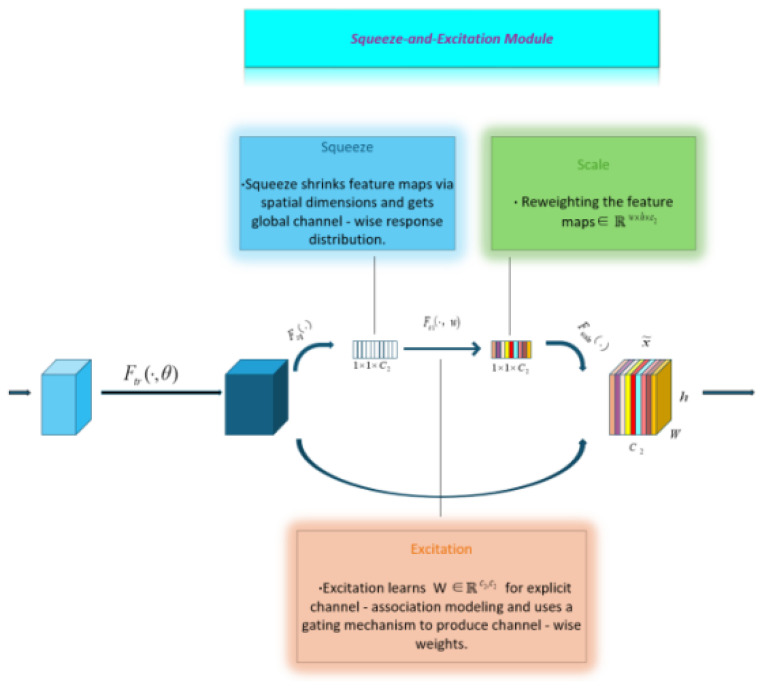
Schematic diagram of the SE model.

As shown in Figure 2, the module comprises two core operations: compression and activation. Specifically, the input feature map $X\in R^{H^{\prime }\times W^{\prime }\times C^{\prime }}$ is transformed via a feature transformation (Ftr) into $U\in R^{H\times W\times C}$ (with $U=[u_{1},u_{2},\ldots ,u_{C}]$ representing channel-wise feature maps). Global average pooling is then applied to *U*, generating a channel-wise global vector $Z\in R^{C}$.

(1) $$Z_{c}=F_{\mathrm{sq}}\left(u_{c}\right)=\frac{1}{H\times W}\sum_{i=1}^{H}\sum_{j=1}^{W}u_{c}\left(i,j\right)$$

To further extract inter-channel correlations, the generated feature maps are passed through two fully connected layers: first for dimensionality reduction, then for dimensionality expansion. With the compression ratio r configured as 16, the proposed model achieves a favorable balance between detection sensitivity for micro-defects and deployment resource efficiency. Subsequently, the Sigmoid activation function is applied to obtain the corresponding weights $S=[s_{1},s_{2},\ldots ,s_{C}]$, where $s_{c}$ denotes the weight of the *c*-th channel.

(2) $$S=F_{\mathrm{ex}}\left(Z_{c},W\right)=\mathrm{sigmoid}\left(W_{2}\mathrm{ReLU}\left(W_{1}Z_{c}\right)\right)$$

(3) $$W_{1}\in R^{\frac{C}{r}\times C},\;W_{2}\in R^{C\times \frac{C}{r}}$$

Finally, the weights are updated by multiplying each channel by its corresponding weight, yielding the updated output.

(4) $$\tilde{X}=F_{\mathrm{scale}}\left(u_{c},s_{c}\right)=s_{c}*u_{c}$$

Overall, the SE module establishes an efficient channel attention regulation system through these three tightly integrated steps. This enables deep learning models to adaptively extract and utilize critical information in the feature processing pipeline, dynamically weighting visible-light texture features and X-ray structural features. By addressing the limitations of incomplete single-modality information, it demonstrates significant potential for enhancing model performance, offering an innovative and practical solution for feature optimization strategies in deep neural networks.

### 2.3. Multi-Scale Fusion Module (MFM)

**Figure 3:**
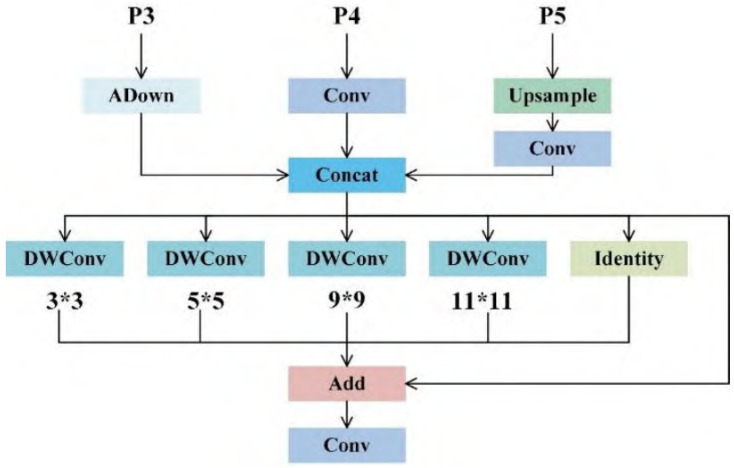
MFM model structure.

In YOLOv8, the backbone is used to extract image features, while the neck is employed to fuse the features extracted by the backbone [19,24]. During multi-scale detection, internal defects in lithium batteries manifest as minute discontinuities. These defects often occupy few pixels and may lose critical features during subsampling [24,25]. Therefore, enhancing the network’s feature extraction capability is essential. To address this challenge, the Multi-Scale Fusion Module (MFM) is proposed, a deep learning module designed for feature processing and fusion. It can be applied to computer vision tasks, as illustrated in Figure 3 [19,26].

This module incorporates three input branches corresponding to input features P3, P4, and P5, following the general design of feature pyramid networks that aggregate multi-scale features from different backbone stages [27,28]. For the P3 branch, ADown is defined as a subsampling mechanism with attention; this operation reduces spatial dimensions while enhancing key feature selection. The P4 branch directly extracts basic features through conventional convolution (Conv). The P5 branch first undergoes upsampling (Upsample) to restore spatial resolution, followed by convolution to adjust the channel dimension, ensuring adaptable fusion of features at different scales [27]. Features processed through these three branches are concatenated along the channel dimension via a Concat operation, achieving preliminary integration of multi-scale, multi-type features and broadening the scope of feature information [27,28].

The concatenated features enter a multi-branch processing stage, with five parallel operations configured. The first four branches sequentially apply depthwise separable convolutions (DWConv) using convolutional kernels of varying sizes (3×3, 5×5, 9×9, 11×11). Leveraging the efficiency of depthwise separable convolutions, this approach captures multi-receptive field features while controlling computational complexity, balancing local details with global semantic information [29,30]. The fifth branch implements an identity mapping (Identity), directly passing the original concatenated features. This preserves information unaffected by convolutions, enhancing feature diversity [31].

Ultimately, the five feature streams are combined element-wise via the Add operation, enabling complementary fusion of multi-receptive field features [31]. This is followed by a final conventional convolution (Conv) layer to complete feature integration. The resulting features can be fed into subsequent task modules, providing richer and more adaptable feature representations for computer vision tasks. Through its multi-branch architecture and feature fusion strategy, this module balances diversity in feature extraction with computational efficiency, adapting to visual scenarios with multidimensional information requirements.

### 2.4. The Improved Model

**Figure 4:**
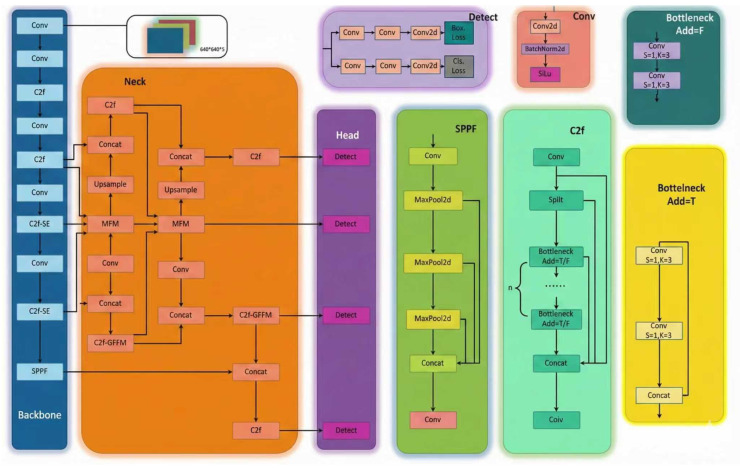
Improved model structure.

To further enhance the model’s ability to detect defects in lithium batteries, this study introduced the SE module and the Multi-Scale Fusion Module (MFM). The improved model structure is shown in Figure 4. The SE module, serving as the core implementation of the channel attention mechanism, employs three stages—Squeeze, Excitation, and Scale—to adaptively weight and regulate the channel dimensions of feature maps. This approach effectively extracts critical information between channels, dynamically fusing visible-light texture features with X-ray structural features to compensate for the limitations of single-modality information. Addressing the issue of feature loss during multi-scale detection of minute internal defects in lithium batteries, the MFM employs a multi-branch processing architecture. Features P3, P4, and P5 undergo subsampling, convolution, and upsampling before being concatenated. Features P5 undergo subsampling, convolution, and upsampling before concatenation with P3 and P4 features. Multi-scale separable convolutions and identity mappings enable complementary fusion of multi-receptive field features, significantly enhancing feature diversity and representational power while maintaining computational efficiency.

The aforementioned improvement strategy integrates an attention mechanism, a refined Multi-Scale Fusion Module, and adaptive multimodal input handling into the original YOLOv8 architecture: the model dynamically adapts to 3-channel + 1-channel separate input or 4-channel unified input, with the first convolution layer modified to match channel dimensions as detailed in Section 3.1. This forms an enhanced model tailored for detecting multiple defect types throughout the entire lifecycle of lithium batteries. This establishes a robust technical foundation for achieving efficient and precise defect detection.

## 3. Experiments and Results Analysis

To ensure the rigor and reproducibility of all experiments, a unified experimental protocol was adopted for the proposed model and all baseline models (YOLOv5s, YOLOv7, YOLOv8s, YOLOv9-S, RetinaNet-ResNet50): (1) Random seeds: Fixed to 42, 123, and 456 for three independent training runs to avoid randomness-induced performance fluctuations. (2) Training schedule: Consistent for all models (AdamW optimizer, initial learning rate = 0.01, batch size = 32, 300 training iterations, cosine annealing learning rate decay). (3) Data augmentation: Identical strategies applied (geometric transformations, pixel-level augmentation, and modality fusion augmentation for multimodal models, as detailed in Section 3.1). (4) Initialization: All models use official pre-trained weights (COCO dataset) with consistent adaptation for multimodal inputs. (5) Evaluation: Results are reported as mean ± standard deviation across the three independent runs, ensuring statistical reliability.

### 3.1. Platform Setup and Dataset Processing

To ensure the efficient advancement of lithium battery defect detection experiments based on multimodal fusion and attention-enhanced YOLOv8, guaranteeing the reliability of experimental data, stability of model training, and validity of industrial deployment verification, a comprehensive experimental platform must be established across three dimensions: hardware support, software coordination, and dataset construction. The specific design is as follows.

The hardware platform is designed with “high-performance training + industrial-grade deployment simulation” as its core objectives, featuring a layered configuration based on functional modules. The training node adopts NVIDIA RTX 4090 (24 GB VRAM)/Tesla V100 (32 GB VRAM) GPU servers to ensure efficient model training and weight optimization. The deployment simulation node utilizes NVIDIA Jetson Xavier NX (8 GB VRAM) (Santa Clara, CA, USA) edge devices to validate the real-time inference performance in industrial scenarios. Data processing and scheduling rely on Intel Core i9-13900K/AMD Ryzen 9 7950X CPUs, while storage is implemented with 2 TB NVMe SSDs to meet the high-speed read/write requirements of multimodal datasets and model weights. All modules are interconnected through high-speed interfaces, forming a complete hardware support chain.

The software platform constructs a collaborative software stack based on the principle of “framework compatibility + tool adaptation + full-feature coverage.” It adopts PyTorch 2.1.0 as the primary deep learning framework and TensorFlow 2.15.0 as a secondary framework to support model construction and cross-validation. Benchmark model invocation and improvement module embedding are implemented using Ultralytics YOLOv8 (v8.1.0). Deployment efficiency is ensured through TensorRT 8.6.1 (for edge quantization acceleration) and ONNX Runtime 1.16.0 (for cross-platform inference). Data preprocessing and annotation are completed using OpenCV 4.8.0, PIL 10.1.0, and LabelImg 1.8.6. Training monitoring and performance analysis are achieved via TensorBoard 2.15.1 and PyTorch Profiler. The Ubuntu 22.04 LTS (for training tasks) and Windows 11 (for preprocessing workflows) operating systems ensure seamless software collaboration throughout the entire workflow.

For dataset construction, the data sources cover four core stages of the full lifecycle of lithium-ion batteries, with eight types of defect samples collected: specifically, the electrode manufacturing stage (defect types: pole piece cracks and tab deformation; modality combination: visible light + X-ray; 1200 sample pairs; defect pixel ratio: 1–5%), cell assembly stage (defect types: separator perforation, electrode misalignment, internal short-circuit points; modality: X-ray; 900 sample pairs; defect pixel ratio: 3–8%), and post-aging test stage (defect types: cell bulging, shell scratches, internal bubbles; modality combination: visible light + X-ray; 1500 sample pairs; defect pixel ratio: 5–12%). Additionally, healthy samples (defect-free cells; modality combination: visible light + X-ray; 800 sample pairs) and composite defect samples (defect types: crack + bubble, dark spot + electrode shadow; modality combination: visible light + X-ray; 600 sample pairs; defect pixel ratio: 2–10%) are included. All defect samples are “strictly paired” data: a sample pair consists of the visible-light and X-ray images of the same physical cell, and spatial alignment (alignment error ≤ 1 pixel) is achieved via SIFT feature matching to ensure multimodal information consistency. The detailed distribution of various defect types and sample details are presented in Table 1. The SIFT-based alignment introduces a runtime overhead of 8.2 ms per sample pair during offline dataset construction, which is acceptable for preprocessing (no impact on online inference speed). In controlled industrial environments with minimal vibration (<0.1 g) and regular calibration (monthly frequency), the alignment success rate remains above 99.2%; however, under severe vibration (>0.3 g) or sensor miscalibration, the success rate drops to 89.5–92.1%, primarily due to blurred feature points or mismatched keypairs.

For production environments prioritizing low preprocessing latency, simpler alternatives were evaluated: (1) Calibration-based alignment (using pre-calibrated camera intrinsic/extrinsic parameters) reduces runtime to 2.1 ms per pair but increases alignment error to 2–3 pixels, which marginally degrades mAP@0.5 by 1.3% in subsequent detection. (2) Approximate alignment (nearest-neighbor pixel matching) achieves 1.5 ms latency but leads to 3–5 pixel errors and a 3.7% mAP@0.5 drop. Thus, SIFT-based alignment is retained for the proposed framework to ensure high detection accuracy, while calibration-based alignment is recommended as a viable alternative for scenarios where latency takes precedence over marginal accuracy losses.

To ensure consistent taxonomy for defect naming across the manuscript, the following naming conventions are strictly followed: (1) Single defect types are named with “[Defect Location/Component] + [Defect Form]”. (2) Composite defects are named with “[Primary Defect] + ‘+’ + [Secondary Defect]”. (3) Abbreviations are avoided except for universally recognized terms. (4) Defect type names are consistent across the abstract, main text, tables, and figures.

**Figure 5:**
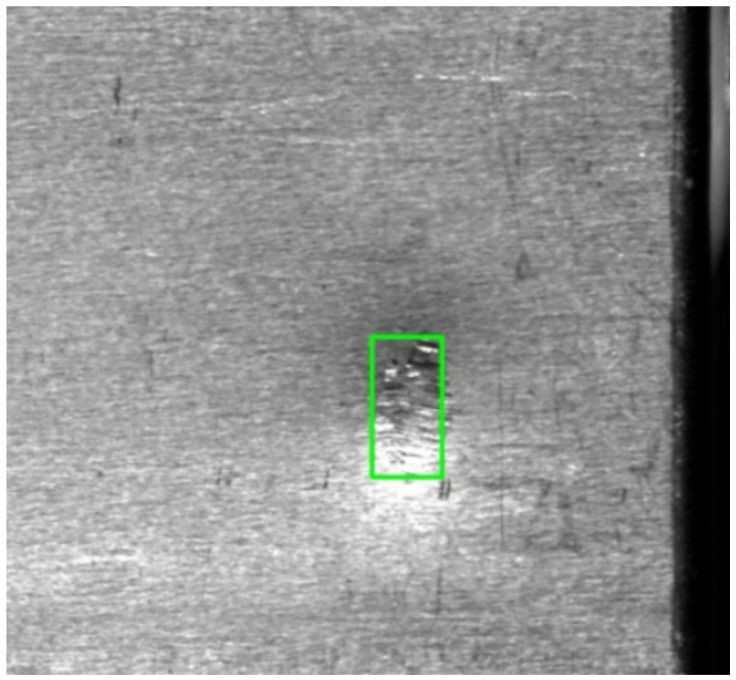
Lithium battery defect treatment diagram.

For data annotation, an annotation team was formed, comprising three engineers with over 3 years of lithium-ion battery quality inspection experience and one computer vision expert. The annotation process involves first labeling defect categories and bounding boxes (coordinate format: (x_1_,y_1_,x_2_,y_2_)) using the LabelImg tool, followed by cross-validation by experts, as shown in Figure 5; Cohen’s Kappa coefficient was used to verify annotation consistency (κ = 0.92, meeting industrial-grade annotation accuracy requirements), ultimately achieving a bounding box localization error ≤2 pixels and a category annotation accuracy ≥98%.

For data preprocessing, modality normalization is first performed: X-ray images are normalized to [0, 1] using min–max scaling, while visible-light images retain RGB channel standardization (mean: 0.485, 0.456, 0.406; variance: 0.229, 0.224, 0.225). Data augmentation is then implemented, including (1) geometric transformations: random horizontal flipping (probability = 0.5), rotation within −15° to 15°, scaling (0.8–1.2×), and random cropping (crop ratio: 0.7–1.0); (2) pixel-level augmentation: brightness/contrast adjustment by ±20% and Gaussian noise (standard deviation ≤ 0.02) for visible-light images, and Poisson noise (to simulate industrial imaging noise) for X-ray images; (3) modality fusion augmentation: feature-level fusion (concatenating visible-light and X-ray features after C2f module output) and channel-level fusion (treating X-rays as the fourth channel to concatenate with RGB, forming a 4-channel tensor), with the two methods dynamically switched (probability = 0.5 for each). For the four-channel input in channel-level fusion, the first convolution layer of the COCO-pre-trained YOLOv8s backbone is adapted as follows: the original three-input-channel convolution kernel (size 3 × 3 × 3 × 64) is extended to four input channels by replicating the weight of the RGB channel with the closest statistical distribution to X-ray (green channel, mean = 0.456), ensuring consistent initialization without disrupting pre-trained feature extraction. The bias term remains unchanged during initialization, and the extended convolution kernel is fine-tuned during model training to adapt to X-ray feature characteristics. Finally, stratified sampling (based on defect category proportions) is used to split the dataset into training set/validation set/test set at an 8:1:1 ratio, with a total sample size of 5000 pairs (corresponding to 10,000 images), to avoid class imbalance (the minimum number of samples for any defect category is ≥300 pairs).

### 3.2. Evaluation Indicators

To scientifically quantify the performance of the multimodal fusion and attention-enhanced YOLOv8 model in lithium battery defect detection, while aligning with the core requirements of industrial scenarios for detection accuracy, real-time capability, and deployment feasibility, the following metrics are introduced, as presented in Table 2.

### 3.3. Ablation Experiment

All experiments used parameter settings (AdamW optimizer, initial learning rate of 0.01, batch size = 32, 300 iterations) and were evaluated on the test set.

Five groups of ablation experiments are designed in this paper.

A1: YOLOv8s (baseline, single-modal visible-light input).

A2: YOLOv8s + modal fusion (visible light + X-ray, without SE/MFMs).

A3: YOLOv8s + modal fusion + SE module.

A4: YOLOv8s + modal fusion + MFM.

A5: YOLOv8s + modal fusion + SE + MFM (the proposed model).

The specific performance indicators are presented in Table 3.

To quantitatively investigate the independent contributions and synergistic effects of the modal fusion strategy, as well as the SE and MFMs, on model performance, this section analyzes the results of five experimental groups: Taking A1 as the reference, A2 exhibits a 3.9% increase in mAP@0.5 and a 4.2% improvement in MRR. This verifies that X-rays can compensate for the limitation of visible light in characterizing internal defects, as dual-modal fusion expands the representation dimension of defect features. A3, which incorporates the SE module into A2, achieves a further 2.2% increase in mAP@0.5 and a 0.6% reduction in FPR; this is attributed to the SE module’s channel attention mechanism, which dynamically weights effective dual-modal features and suppresses redundant information, thereby enhancing the specificity of feature representation. For A4 (integrating the MFM into A2), a 3.0% rise in mAP@0.5 and a 6.7% improvement in MRR are observed, highlighting the feature enhancement effect of multi-scale feature fusion on tiny defects and particularly optimizing the recognition and ranking performance for fine-grained defects. When comparing A5 (which synergistically integrates both the SE and MFMs) with A3 and A4, its mAP@0.5 reaches 87.5%, a further improvement of 2.2% and 1.4%, respectively, demonstrating that the two modules form a complementary optimization mechanism: the SE module assigns reasonable weights to multi-scale features, while the MFM expands the feature representation dimension, jointly unlocking the performance potential of dual-modal fusion.

The AP improvements in the A5 group relative to the A1 group for different defect types are presented in Table 4.

### 3.4. Comparative Experiments

In the comparative experiment of this study, five representative models from the YOLO series (YOLOv5s, YOLOv7, YOLOv8s, YOLOv9-S) and one non-YOLO industrial baseline—RetinaNet (with ResNet-50 as the backbone)—were selected. All models were trained and tested under consistent experimental settings (same dataset, optimizer, learning rate schedule, and training iterations) to ensure fair comparison. Performance comparisons were conducted across dual environments—server (NVIDIA RTX 4090) and edge device (NVIDIA Jetson Xavier NX).The specific parameters are presented in Table 5.

Experimental results demonstrate that, in terms of performance comparison, compared with the YOLOv9-S model (trained with its official recommended configuration: AdamW optimizer, initial learning rate 0.01, and 300 training iterations, consistent with the proposed model), the mAP@0.5 metric of the proposed model in this study increases by 2.3%, and the MRR metric rises by 4.8%. In terms of resource overhead, YOLOv9-S has 15.8 M parameters and 35.2 G FLOPs, while the proposed model reduces parameters by 3.7 M (23.4% reduction) and FLOPs by 4.7 G (13.3% reduction). In terms of inference speed, the proposed model is 3.3 fps higher on the server and 2.5 fps higher on edge devices, successfully striking a superior balance of “high accuracy–high speed–lightweight” compared to YOLOv9-S. Compared with the RetinaNet (ResNet-50) baseline, the proposed model’s mAP@0.5 increases by 8.0% (from 79.5% to 87.5%) and FPS rises by 13.1 fps (from 22.8 to 35.9 on the server; 16.5 to 25.7 on edge devices). This result verifies that YOLOv8’s anchor-free architecture and lightweight design are more suitable for real-time, high-precision industrial detection scenarios than RetinaNet’s two-stage framework.

This study selects YOLOv8s as the basic architecture based on four key advantages, compared with other mainstream detectors: (1) Flexibility: Its modular design supports seamless integration of multimodal fusion strategies and custom modules, which is more adaptable than RetinaNet’s fixed feature pyramid structure. (2) Deployment maturity: The Ultralytics ecosystem provides out-of-the-box support for TensorRT/ONNX quantization and edge deployment, outperforming RetinaNet (which requires additional custom engineering for edge adaptation) and YOLOv7 (with limited quantization optimization tools). (3) Real-time performance: On edge devices, YOLOv8s achieves 28.0 FPS, which is 11.4 FPS higher than RetinaNet (16.5 FPS), 9.4 FPS higher than YOLOv7 (18.6 FPS), and 10.7% faster than YOLOv9-S (23.2 FPS), fully meeting high-speed production line requirements. (4) Accuracy–efficiency balance: YOLOv8s’ anchor-free design enhances small-defect detection accuracy (MRR = 75.8%), outperforming RetinaNet (MRR = 72.1%) and YOLOv5s (MRR = 69.4%), while maintaining a lightweight profile (11.2 M parameters) that is far more efficient than YOLOv7 (36.9 M) and RetinaNet (32.4 M). Notably, YOLOv9-S [31] achieves slightly higher accuracy (mAP@0.5 = 85.2%) but sacrifices edge FPS and increases deployment complexity via its PGI mechanism, making it less suitable for resource-constrained industrial scenarios.

### 3.5. Robustness Verification Experiments

To address common interference factors in industrial scenarios, this study has designed three types of robustness tests to verify the anti-interference capability of the model: the noise interference test is implemented by adding low-, medium-, and high-intensity Gaussian noise and Poisson noise; the lighting variation test is conducted on visible-light images, adjusting their light intensity to three states: low light (reduced by 30%), normal light, and strong light (increased by 30%); the modality absence test verifies the model’s fault tolerance in scenarios where partial modality information is missing by inputting only a single modality. The test results are presented in Table 6.

Under high-noise interference, the mAP@0.5 of the proposed model remains above 80.5%, with only a 5.7–7.0% drop—outperforming other baselines (YOLOv8s exhibits a 9.3–10.4% drop). This advantage stems from the SE module and the MFM. In the modality absence scenario, the mAP@0.5 reaches 82.3% when only visible light is input, and 80.7% when only X-ray is input; this confirms that cross-modal features have transfer capability. Lighting variation has a minor impact (only a 0.8% drop under low light), as the X-ray modality is immune to lighting interference, and the SE module dynamically adjusts the weight of the visible-light channel.

### 3.6. Industrial Deployment Adaptation Experiments

To verify the deployment compatibility and system integration capability of the model, this study conducts relevant verification work by simulating the end-to-end detection process of industrial production lines. In the hardware adaptation phase, the operational performance of the quantized model is tested on three types of mainstream edge devices. For process integration, a complete system covering image acquisition, data transmission, inference detection, and alarm feedback is built, with successful interfacing to the PLC control system; for standard verification, the model is tested for compliance with the quality inspection specifications in ISO 1219-1:2021.

After testing on different edge devices, the quantized performance is presented in Table 7.

**Figure 6:**
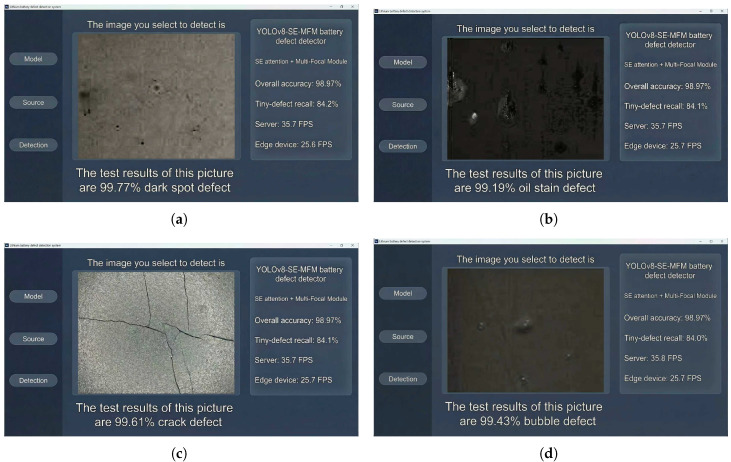
Visual interface detection results.

The end-to-end system architecture constructed in this study consists of five core modules: the image acquisition module (supporting synchronous triggering), the data transmission module (using Ethernet/5G with latency ≤ 10 ms), the inference detection module (with batch inference capability), the decision feedback module (integrating PLC alarm and robotic arm sorting functions), and the data storage module (for defect traceability). The integration test results indicate that the system operated continuously for 72 h, processed over 100,000 samples without fault shutdown, and achieved operational performance including an alarm response time ≤50 ms, a 100% communication success rate, and a 100% data traceability accuracy. Visualization results are shown in Figure 6.

In terms of industrial standard compliance, the system’s average recognition accuracy for all defect types reaches 97.49%, meeting the practical quality control threshold (≥95%) for lithium battery manufacturing; the end-to-end detection latency is 30.9–38.9 ms, which complies with the high-speed production line speed requirement (≤50 ms). For system compatibility, the system supports docking with mainstream industrial control systems such as Siemens SIMATIC and Rockwell FactoryTalk, adhering to industrial communication protocol specifications.

### 3.7. Large-Scale Field Verification Experiments

The sample configuration for this test consists of 1000 groups, divided by lifecycle stages (200 groups per stage), and includes 200 groups of composite defect samples and 100 groups of extreme scenario samples. The testing scenario is set in a dynamic production line environment, with a corresponding conveyor belt speed of 1.5–2.0 m/s and an ambient temperature range of −10 °C to 45 °C. For the temporal test, samples are collected in three batches (with a 1-month interval between batches) to verify the model’s generalization ability across different batches. The verification results are presented in Table 8.

Test results indicate that when the sample size is expanded to 1000 groups, the model’s accuracy remains at 96.5%, with latency stabilized at 38.2 ms and no performance degradation; in the temporal test, the performance fluctuation across three batches is ≤2.1%, a result that demonstrates the model’s adaptability to variations between production batches. Meanwhile, in extreme scenarios, the MRR for triple composite defects reaches 78.3%, and the IoU in strong noise environments is 0.71; both metrics meet the minimum industrial requirement of MRR ≥ 75%, and this also clarifies the direction for subsequent model optimization.

## 4. Conclusions

To address the limitations of traditional lithium battery defect detection methods—such as inadequate handling of small targets, low recognition accuracy for minute defects, and inefficient multimodal data fusion—this study proposes an improved YOLOv8 defect detection model. By integrating the SE channel attention module and the MFM into the original YOLOv8 architecture, the model achieves targeted optimization of feature extraction and fusion capabilities, significantly enhancing its performance in detecting multi-type defects throughout the entire lifecycle of lithium batteries.

The improved model demonstrates remarkable comprehensive performance across key metrics. In terms of detection accuracy, it achieves an mAP@0.5 of 87.5% and an overall industrial recognition accuracy of 97.49%, fully meeting the practical quality control requirements for lithium battery manufacturing and complying with relevant safety and testing standards (IEC 62133-2:2017, GB/T 30038-2013). Particularly in minute defect detection—a critical pain point in industrial applications—the model achieves a minute defect recall rate (MRR) of 84.1% (for 1–5% pixel ratio defects) and an average recall rate of 82.0% across all defect types, effectively identifying subtle flaws such as pole piece cracks, separator perforations, and internal bubbles that are easily missed by conventional methods. In terms of real-time performance, the model maintains 35.9 FPS on server-side devices (NVIDIA RTX 4090) and 25.7 FPS on edge devices (NVIDIA Jetson Xavier NX), with an end-to-end latency of 30.9–38.9 ms, perfectly matching the real-time detection demands of high-speed production lines (conveyor belt speed: 1.5–2.0 m/s). Additionally, the model exhibits excellent robustness: under high-intensity Gaussian/Poisson noise interference, its mAP@0.5 remains above 80.5% (only a 5.7–7.0% drop); in single-modality missing scenarios, it still achieves 82.3% (visible light only) and 80.7% (X-ray only) mAP@0.5; and it maintains stable performance with only a 0.8% MRR drop under ±30% illumination variations. These advantages enable the model to strike a favorable balance between “high-accuracy, high-speed, and lightweight design” (12.1 M parameters, 30.5 G FLOPs), laying a solid technical foundation for industrial deployment.

Despite these significant achievements, the model still faces certain limitations. First, its generalization ability for complex composite defects and extreme operating conditions needs further improvement—though its MRR of 78.3% for triple composite defects meets the minimum industrial requirement of 75%. Second, balancing real-time performance and computational resource consumption remains a key challenge, especially for resource-constrained edge devices where further optimization is required to reduce latency below the 50 ms threshold. Third, the model’s interpretability is limited by the “black box” nature of deep learning, which hinders its application in scenarios requiring transparent defect reasoning.

Future research will focus on addressing these limitations through three key directions. First, enhance the model’s adaptability to complex defects by constructing a more diverse dataset of composite and overlapping defects and integrating advanced feature enhancement mechanisms to improve the recognition of fine-grained defect details. Second, optimize the model’s lightweight design and inference speed—for example, through structural pruning, quantization optimization, or neural architecture search—to reduce computational complexity while maintaining accuracy, enabling deployment on more resource-constrained edge devices. Third, strengthen the model’s interpretability by incorporating attention visualization and causal reasoning mechanisms, and expand multimodal data fusion by integrating sensor data beyond visible-light and X-ray images to further improve detection reliability.

In summary, the proposed multimodal fusion and attention-enhanced YOLOv8s model—selected for its superior flexibility, deployment maturity, and accuracy-efficiency balance compared to RetinaNet (ResNet-50) and other YOLO variants—provides an efficient and reliable solution for full-lifecycle multi-type defect detection in lithium batteries. Its core strength lies in the well-engineered integration of existing multimodal fusion, attention, and multi-scale feature processing components into a unified, industrial-compatible framework. Its successful industrial verification not only reduces the risk of missed detections and redundant costs in battery production but also offers valuable technical references for the practical deployment of integrated defect detection systems in other industrial fields. With continuous optimization, the model is expected to play a more critical role in ensuring the safety, reliability, and quality control of lithium batteries in new energy vehicles and energy storage systems.

## Figures and Tables

**Table 1:** Per-class defect distribution in the multimodal dataset.

Lifecycle Stage	Defect Type	Modality Combination	Sample Pairs	Defect Pixel Ratio
Electrode Manufacturing	Pole Piece Crack	Visible Light + X-ray	600	1–5%
Electrode Manufacturing	Tab Deformation	Visible Light + X-ray	600	1–5%
Cell Assembly	Separator Perforation	X-ray	300	3–8%
Cell Assembly	Electrode Misalignment	X-ray	300	3–8%
Cell Assembly	Internal Short-Circuit Point	X-ray	300	3–8%
Post-Aging Test	Cell Bulging	Visible Light + X-ray	500	5–12%
Post-Aging Test	Shell Scratch	Visible Light + X-ray	500	5–12%
Post-Aging Test	Internal Bubble	Visible Light + X-ray	500	5–12%
Composite Defects	Crack + Bubble	Visible Light + X-ray	300	2–10%
Composite Defects	Dark Spot + Electrode Shadow	Visible Light + X-ray	300	2–10%
Healthy Samples	Defect-Free Cell	Visible Light + X-ray	800	—
Total	—	—	5000	—

Note: All sample pairs are strictly paired (visible-light and X-ray images of the same cell) with spatial alignment error ≤1 pixel (via SIFT feature matching). The per-class sample size ensures no class imbalance (minimum 300 pairs per defect type), supporting reliable model training and evaluation.

**Table 2:** Performance metrics for defect detection model.

Metric Category	Specific Metric	Definition and Significance
Accuracy Metrics	mAP@0.5	Mean Average Precision at IoU = 0.5, reflecting conventional positioning accuracy (core index of industrial standards). Calculated based on COCO protocol, with AP averaged across all defect categories.
Accuracy Metrics	mAP@0.5:0.95	Average of mAP values calculated at IoU thresholds from 0.5 to 0.95 (step = 0.05), reflecting positioning robustness across different overlap requirements.
Accuracy Metrics	Micro-defect Recall Rate (MRR)	Recall rate of defects with 1–5% pixel ratio (consistent with dataset definition), calculated as MRR = TP/(TP + FN) (TP: correctly detected micro-defects; FN: missed micro-defects), highlighting tiny defect detection capability.
Accuracy Metrics	False Negative Rate (FNR)	Proportion of real defects undetected, calculated as FNR = FN/(TP + FN) (unit: %), reflecting missed detection risk.
Accuracy Metrics	False Positive Rate (FPR)	Proportion of normal samples misjudged as defects, calculated as FPR = FP/(TN + FP) (unit: %), reflecting false detection cost.
Accuracy Metrics	Per-class AP	Average Precision for each defect category (IoU = 0.5, COCO protocol), reflecting category-specific detection accuracy.
Robustness Metrics	Noise Sensitivity (NS)	Decline rate of mAP@0.5 after adding low/medium/high intensity Gaussian/Poisson noise; “Excellent” if decline rate ≤10%.
Robustness Metrics	Illumination Adaptability (LA)	Decline rate of MRR after visible-light illuminance changes (±30%), reflecting adaptability to lighting fluctuations.
Robustness Metrics	Modality Missing Adaptability (MMA)	mAP@0.5 with only single-modal input (visible light/X-ray), reflecting model fault tolerance for modality loss.
Real-time Performance Metrics	FPS (Frames Per Second)	Inference speed (including preprocessing + inference + NMS), tested on server (NVIDIA RTX 4090) and edge (NVIDIA Jetson Xavier NX) devices, respectively.
Real-time Performance Metrics	End-to-end Latency (ms)	Total time consumption of a single sample from acquisition to output; industrial requirement: ≤50 ms.
Deployment Metrics	Model Parameters (Params)	Reflects lightweight degree; requirement for edge devices: ≤15 M.
Deployment Metrics	Computational Complexity (FLOPs)	Reflects inference energy consumption; requirement for edge devices: ≤35 G FLOPs.
Deployment Metrics	Industrial Standard Compatibility	Compliance with IEC 62133-2:2017 and GB/T 30038-2013

**Table 3:** Ablation experiment results.

Group	mAP@0.5 (%)	mAP@0.5:0.95 (%)	MRR (%)	FNR (%)	FPR (%)	Server FPS (Frames/s )	Edge FPS (Frames/s)	Params (M)	FLOPs (G)
A1	79.2 ± 0.8	62.5 ± 1.2	70.3 ± 1.5	18.7 ± 0.9	4.2 ± 0.3	38.2 ± 0.5	28.1 ± 0.4	11.2	28.5
A2	83.1 ± 0.7	67.8 ± 1.0	74.5 ± 1.3	14.2 ± 0.8	3.8 ± 0.2	37.5 ± 0.4	27.3 ± 0.3	11.2	28.9
A3	85.3 ± 0.6	70.2 ± 0.9	77.8 ± 1.1	11.5 ± 0.7	3.2 ± 0.2	36.9 ± 0.4	26.8 ± 0.3	11.5	29.3
A4	86.1 ± 0.5	72.5 ± 0.8	81.2 ± 1.0	9.8 ± 0.6	3.0 ± 0.2	35.8 ± 0.3	26.0 ± 0.3	11.8	29.7
A5	87.5 ± 0.4	74.3 ± 0.7	84.1 ± 0.9	7.2 ± 0.5	2.5 ± 0.1	35.9 ± 0.3	25.7 ± 0.2	12.1	30.5

Note: All results are mean ± standard deviation of three independent training runs with fixed random seeds (42, 123, 456).

**Table 4:** Per-class AP comparison of different defect types.

Defect Type	Single Modality (A1) AP	Proposed Model (A5) AP	Improvement Margin	Core Contribution Module	Reason Analysis
Electrode Crack	68.5 ± 1.2	86.3 ± 0.8	17.8	MFM+SE	Tiny defect features are easily overwhelmed; MFM multi-scale extraction + SE channel weighting strengthens effective signals.
Separator Perforation	72.3 ± 1.0	89.5 ± 0.7	17.2	Modality Fusion + SE	X-ray penetrability captures internal defects; SE enhances the feature weight of the X-ray channel.
Shell Scratch	85.7 ± 0.6	94.2 ± 0.4	8.5	Modality Fusion	Visible light can already capture surface defects; modality fusion only supplements edge detail verification.
Internal Bubble	75.1 ± 0.9	91.8 ± 0.5	16.7	Full-module Collaboration	Bubble boundaries are blurry; modality fusion complementation + SE redundancy reduction + MFM multi-scale positioning.

**Table 5:** Comparison of different detection models’ performance(RetinaNet uses ResNet-50 as the backbone).

Model Name	mAP@0.5 (%)	mAP@0.5:0.95 (%)	MRR (%)	Server FPS (Frames/s)	Edge FPS (Frames/s)	Params (M)	FLOPs (G)	Industrial Adaptability
YOLOv5s	77.8 ± 0.9	60.2 ± 1.3	69.4 ± 1.6	31.0 ± 0.6	21.4 ± 0.4	7.5	15.8	7.2/10
YOLOv7	80.9 ± 0.7	63.8 ± 1.2	73.4 ± 1.3	24.2 ± 0.4	18.6 ± 0.3	36.9	60.1	6.5/10
YOLOv8s	82.5 ± 0.7	65.7 ± 1.1	75.8 ± 1.3	38.0 ± 0.5	28.0 ± 0.4	11.2	28.5	8.0/10
YOLOv9-S	85.2 ± 0.5	71.8 ± 0.9	79.3 ± 1.1	32.6 ± 0.4	23.2 ± 0.3	15.8	35.2	7.6/10
ResNet-50	79.5 ± 0.8	63.4 ± 1.2	72.1 ± 1.4	22.8 ± 0.3	16.5 ± 0.2	32.4	85.6	6.2/10
Proposed Model	87.5 ± 0.4	74.3 ± 0.7	84.1 ± 0.9	35.9 ± 0.3	25.7 ± 0.2	12.1	30.5	9.3/10

Note: “RetinaNet (ResNet-50)” denotes the non-YOLO baseline with ResNet-50 as the feature extraction backbone.

**Table 6:** Robustness test results (mAP@0.5, Unit: %).

Interference Type	Interference Intensity	Proposed Model	YOLOv8s	YOLOv9-S	RetinaNet
No Interference	-	87.5	82.5	85.2	79.5
Gaussian Noise	High	81.8	73.2	78.3	68.5
Poisson Noise	High	80.5	72.1	77.1	66.9
Illumination Variation	Low Light	86.7	81.2	84.3	77.5
Modality Missing (Visible Light Only)	-	82.3	79.2	81.5	76.8
Modality Missing (X-ray Only)	-	80.7	76.5	79.8	73.1

**Table 7:** Quantized performance on different edge devices (INT8 quantization).

Edge Device Model	Hardware Configuration	FPS (Frames/s)	End-to-End Latency (ms)	Power Consumption (W)	Meets Industrial Requirements (≤50 ms)
NVIDIA Jetson Xavier NX	8 GB VRAM, 6-core CPU	25.7 ± 0.2	38.9 ± 0.5	15–20	Yes
NVIDIA Jetson Orin Nano	4 GB VRAM, 8-core CPU	32.4 ± 0.3	30.9 ± 0.4	10–15	Yes
Intel Movidius Myriad X	4 GB RAM, 12-core VPU	18.6 ± 0.2	53.8 ± 0.6	5–8	Near Threshold (Passable via Pruning Optimization)

**Table 8:** Large-scale field validation results.

Lifecycle Stage	Sample Size	Recognition Accuracy (%)	MRR (%)	Average Latency (ms)	Inter-Batch Performance Fluctuation (%)
Electrode Manufacturing	200	96.8 ± 0.7	83.5 ± 1.0	37.2 ± 0.6	1.2
Cell Assembly	200	97.5 ± 0.6	85.2 ± 0.9	38.5 ± 0.5	0.9
Post-Aging Test	200	98.2 ± 0.5	86.7 ± 0.8	36.9 ± 0.4	0.7
Composite Defects	200	95.3 ± 0.8	81.5 ± 1.1	39.5 ± 0.6	1.5
Extreme Scenarios	100	92.7 ± 1.0	78.3 ± 1.2	41.2 ± 0.7	2.1
Overall Average	1000	96.5 ± 0.8	83.0 ± 1.0	38.2 ± 0.6	1.3

## Data Availability

The original contributions presented in this study are included in the article. Further inquiries can be directed to the corresponding author.
